# Portable experimental hut tents (PEHTs): a conceptual framework for improved malaria vector control decision-making with a review of fixed experimental hut sites in Africa

**DOI:** 10.1186/s13071-026-07483-1

**Published:** 2026-06-26

**Authors:** Richard M. Oxborough, Trishan Wickramasinghe, Nikita Gopakumar, Michael Fong, Abdul Rahim Mohammed Sabtiu, Whitney A. Qualls, Joseph W. Diclaro, Jaslyn Stamey, Rui-de Xue, Yafet Tiruha, Thomas S. Churcher, Seungman Park, Yaw Afrane, Louisa A. Messenger

**Affiliations:** 1 Parasitology and Vector Biology (PARAVEC) Laboratory, School of Public Health, University of Nevada, Las Vegas, Las Vegas, NV USA; 2 Department of Biological Sciences, College of Southern Nevada, Las Vegas, NV USA; 3 Department of Environmental and Global Health, School of Public Health, University of Nevada, Las Vegas, Las Vegas, NV USA; 4 Department of Mechanical Engineering, Howard R. Hughes College of Engineering, University of Nevada, Las Vegas, Las Vegas, NV USA; 5 Department of Art, University of Nevada, Las Vegas, Las Vegas, NV USA; 6 Department of Medical Microbiology, College of Health Sciences, University of Ghana, Accra, Ghana; 7 Anastasia Mosquito Control District, St. Augustine, FL 32092 USA; 8 School of Public Health, Imperial College London, London, UK

**Keywords:** Portable experimental hut tent (PEHT), Malaria vector control, Insecticide-treated net (ITN), Insecticide resistance, West African-style hut, Subnational tailoring

## Abstract

**Background:**

Insecticide-treated nets (ITNs) remain the primary vector control method across sub-Saharan Africa to prevent malaria transmission, with 2.2 billion pyrethroid (PY) ITNs distributed since 2004. Widespread PY resistance has contributed to stalling progress in recent years; however, new ITNs containing the pyrrole, chlorfenapyr, or the synergist piperonyl butoxide can provide improved efficacy. With more vector control options but limited resources, malaria programs face complex decisions influenced by local transmission intensity, mosquito species, and dynamic insecticide resistance patterns. Decisions are often based on subnational bioassay data, which are not indicative of ITN field performance of newer non-neurotoxic insecticides.

**Methods:**

The gold-standard entomological method for assessing ITN performance is experimental hut trials; however, hut sites are fixed in place and often not representative of high-malaria-burden contexts. This manuscript consists of two components: the first is a literature review of fixed experimental hut sites in Africa. The second describes a new conceptual framework and design of portable experimental hut tents (PEHTs).

**Results:**

The literature review identified 34 experimental hut sites in malaria-endemic Africa. The West-African style hut was the most widely adopted design, used in 24 sites, with most (19/34) located near rice fields for consistent vector yield. The novel PEHT design is based on West African-style huts, using large cabin-style tents equipped with a veranda and 3D-printed lightweight mosquito entry points. A major advantage of PEHTs is their portability for use within high-malaria-burden villages to evaluate community-used ITNs of varying ages against local malaria vector populations, which can support data-driven subnational vector control tailoring. Beyond the evaluation of ITNs, PEHTs can be utilized for studies of new vector control technologies, such as spatial repellent emanators, and can generate evidence for improved vector control practices during humanitarian emergencies, military deployments, and in settings with emerging vector-borne disease threats.

**Conclusions:**

This study describes the innovative concept and design of PEHTs for expanded evaluation of vector control tools in a wider geographical range against local, heterogeneous mosquito populations.

**Graphical Abstract:**

**Figure:**
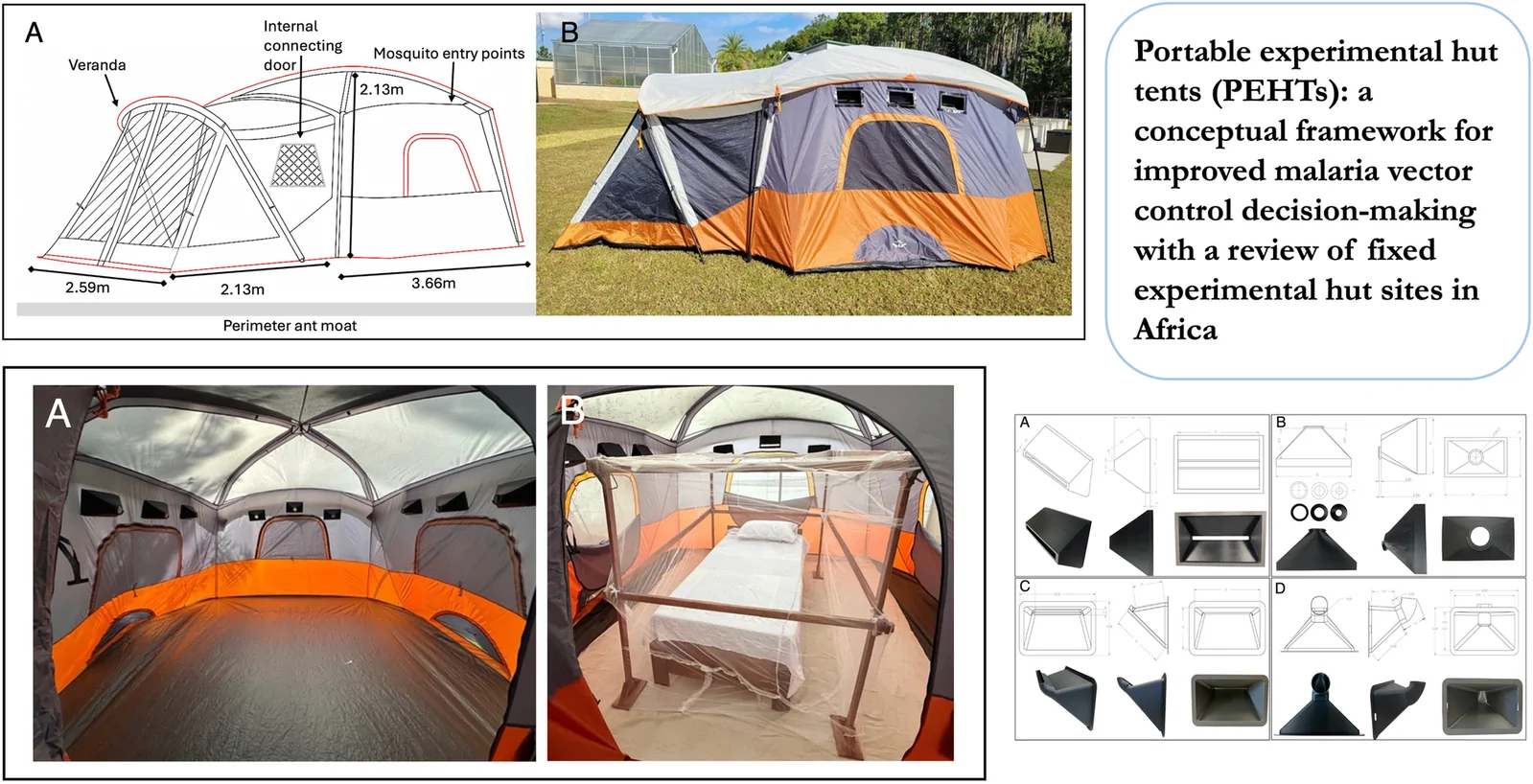

## Background

Insecticide-treated nets (ITNs) are widely used across sub-Saharan Africa, and it is estimated that between 2000 and 2015, vector control programs averted 663 million clinical malaria cases, with ITNs contributing to 68% of that reduction [1]. More recent estimates indicate that between 2000 and 2025, 1.57 billion malaria cases and 6.2 million deaths were averted, driven primarily by vector control [2].

Pyrethroid (PY) insecticides were the dominant chemical class used on ITNs, with an estimated 2.2 billion PY ITNs delivered in sub-Saharan Africa from 2004 to 2025 [3, 4]. While the global malaria mortality rate was halved between 2000 and 2015, progress has stalled and even begun to reverse in recent years, coinciding with growing PY resistance and plateauing intervention coverage [2], and exacerbated by disruptions during the COVID-19 pandemic [5]. PY resistance is ubiquitous and entrenched across sub-Saharan Africa, with high-intensity resistance reported in many countries, following decades of agricultural and vector control use [6–9]. Projections indicate that the 2030 Global Technical Strategy malaria incidence targets may be missed by a staggering 89% [5]. This prolonged period of stagnating progress is deeply concerning, and it is universally acknowledged that improved vector control is essential to tackle the insecticide resistance crisis and to reinvigorate progress toward global malaria elimination.

In recent years, ITNs containing the synergist piperonyl butoxide (PBO) or the non-neurotoxic pyrrole chlorfenapyr (CFP) have proven to be superior to PY ITNs in locations with high-intensity PY resistance [10–13]. The World Health Organization (WHO) strongly recommends deployment of PY-CFP over PY ITNs in areas where mosquitoes have become PY-resistant, and provides a conditional recommendation for PY-PBO ITNs over PY ITNs [14]. The market share for PY-PBO ITNs in sub-Saharan Africa has increased substantially in recent years, with 498 million delivered from 2018 to 2025, reaching a peak proportion in 2023 at 58% (112.6 of 195.4 million) of ITNs delivered [3, 4]. Similarly, delivery of dual-active-ingredient (AI) ITNs (presumed to be mostly PY-CFP ITNs^1^) has increased markedly, with 366 million ITNs delivered from 2019 to 2025, increasing to 74% of ITNs in 2025 (184.5 of 250.5 million) [3].

For more than 20 years, PYs were the only insecticide option for ITNs. However, it is increasingly recognized that a “*one-size-fits-all*” approach is unsustainable and that subnational tailoring of interventions is essential if we are to make progress toward malaria elimination [15]. With greater ITN choice, national malaria control/elimination programs (NMC/EPs) face new critical vector control decisions, which are complicated by regional or localized variation in climate, ecology, malaria transmission intensity, predominant mosquito vector species (characterized by markedly different behaviors), and dynamic insecticide resistance trends and intensities. While PY-PBO ITNs have been distributed in vast numbers in recent years, there is a particularly high risk of mosquitoes overcoming the synergistic effects of PBO given the existing high intensity of PY resistance and multiple, overlapping cross-resistance mechanisms present in many countries. Of great concern is a recent finding in Tanzania that PY-PBO ITNs intensified permethrin resistance 56-fold while multiple resistance mechanisms diminished the synergistic effect of PBO during a 3-year period [16]. Meanwhile, despite limited agricultural use, CFP resistance has already been detected in Cameroon, with underlying mechanisms described [17]. This highlights the dynamic nature of insecticide resistance and the importance of subnational monitoring of ITNs to guide effective and preemptive vector control decision-making.

Cost is a critical limiting factor: PY-PBO ITNs ($2.29) are 17% more expensive and PY-CFP ITNs ($2.84) 46% more expensive than PY ITNs ($1.95) based on 2025 Global Fund Pooled Procurement Mechanism cost estimates [18]. Given the large population at risk of malaria in sub-Saharan Africa and the relatively short lifespan of ITNs (often less than 3 years) [19], a complete switch from PY to PY-CFP ITNs equates to an additional $185 million per year required in stakeholder funding to sustain current distribution targets (mean 214.6 million ITNs delivered per year 2020–2025) [4]. In response to the PY resistance crisis and funding constraints, many countries in sub-Saharan Africa have pragmatically adopted a regional geographic mosaic of PY, PY-PBO and PY-CFP ITNs [20].

Vector control decision-making is often based on insecticide susceptibility and ITN cone bioassay data. Susceptibility bioassays have already shown that PBO does not fully synergize with some PY insecticides in more than 13 countries in sub-Saharan Africa [7, 21–24]. However, there are substantial limitations with such bioassay data, as there is little basis for deciding what threshold of mortality in PY-PBO susceptibility tests indicates likely loss of protective efficacy under field conditions, nor how variable, incomplete PBO synergy corresponds to key entomological outcomes, including blood-feeding inhibition and deterrence. Furthermore, growing evidence indicates that cone bioassays of CFP are unsuitable and not indicative of ITN operational field performance [25–27]. This is because CFP has a non-neurotoxic mode of action, requiring activation during insect cellular respiration, and must instead be evaluated when mosquitoes are in flight and metabolically active [25–28].

Experimental hut trials are the gold-standard entomological method for assessing ITN performance but have been limited by use in fixed locations. Portable designs have been developed such as wooden huts in Belize (Central America) to recapture marked mosquitoes released at different distances, and consisted of a collapsible aluminum frame, allowing the roof, gables, and walls to be dismantled and relocated using transporter trucks [29]. Ifakara huts (Tanzania) were also designed to be portable based on a kit format but have largely been utilized in fixed locations due to their bulky design [30]. An alternative approach to fixed-location experimental huts is the use of lightweight tents that can be transported in the back of a pickup truck. Tents have been used for vector control and surveillance activities on several continents, including insecticide-treated tents in refugee camps [31], odor-baited choice studies [32], and mosquito surveillance of outdoor-biting vectors with the Furvela tent trap [33], Ifakara tent trap [34], Malaise trap [35], baited conventional tents [36], battery-powered Infoscitex tent [37], and the MosqTent [38]. To our knowledge, tents have not been utilized previously for experimental hut studies. Portable experimental hut tents (PEHTs) are a novel evaluation system with expanded utility over traditional fixed-location experimental huts for localized assessment of malaria vector control tool performance.

This manuscript consists of two components. The first is a non-systematic literature review of experimental hut sites, building on Nash et al. [39], to identify the location and characteristics (hut design, primary malaria vector species, ecological characteristics, etc.) of fixed experimental hut sites in Africa used for mosquito vector control or behavior studies conducted from 2000 to 2025. The second describes a new concept and design of PEHTs prior to subsequent optimization and validation experiments. WHO states that “*hut designs may be adapted, or novel designs may be used in different settings. In such cases, appropriate baseline information should be generated to characterize the suitability of the design to support interpretation of data generated in studies using the new/adapted huts*” [40]. A prototype PEHT design is reported in this manuscript. Before PEHTs can be utilized for field evaluations of vector control products, they will be optimized using release–recapture experiments in screenhouses, followed by semi-field studies in reference to fixed West African-style huts.

## Methods

### Challenges of conventional experimental hut studies

Experimental hut trials are the gold-standard entomological method for assessing ITN performance. Fixed-location experimental huts have been used for decades as a standardized WHO system for evaluating the performance of new insecticides (used for both ITN and indoor residual spray [IRS] products) against wild, free-flying *Anopheles* mosquitoes, with experimental hut findings reviewed by the WHO prequalification program (formerly the WHO Pesticide Evaluation Scheme [WHOPES]) for translation into best vector control practices and policies [40–42]. There are several designs of experimental huts used in sub-Saharan Africa that are described by WHO, including East African-style, Ifakara, Rapley, and West African-style huts, which have design features in common [40]. In experimental huts, anthropophagic and endophagic mosquitoes are attracted to concentrated odor plumes emanating from hut eaves. Vectors are allowed unrestricted ingress through entry points to facilitate the collection of an estimable proportion of mosquitoes that interact with the intervention under evaluation, with entry points often baffled to prevent mosquito exit [43, 44]. Recent work has validated how entomological metrics quantified in experimental huts, combined with mechanistic models of malaria transmission, can reliably predict the epidemiological impact of mass use of different types of ITNs assessed in cluster-randomized controlled trials (cRCTs) [45, 46]. However, experimental huts suffer from several logistical and financial constraints. Experimental huts have primarily been used for initial ITN or IRS product registration and due to their fixed locations have rarely been used as a tool to evaluate the ongoing performance of field-used ITNs in communities against their local, heterogeneous mosquito vector populations [28].

Conventional experimental huts are built using robust materials and reused for multiple trials of vector control insecticides and product iterations over several decades in the same fixed locations. Conventional experimental hut construction is relatively expensive, estimated in Tanzania at USD $4000–8000 per hut in 2015, depending on design [47]. Using World Bank annual consumer price inflation data, we estimate a cumulative increase in price levels of approximately 45% in Tanzania (2015–2025) [48], with hut construction costs of USD $5800–11,600 per hut in 2025. With the increasing number of ITN products and recognition of the need for technical replication, trials often utilize 10–15 huts simultaneously, meaning land purchase and infrastructure become costly (depending on location and hut style) [49] and can become obsolete if local breeding sites become unproductive or if a shift in species occurs.

**Fig. 1:**
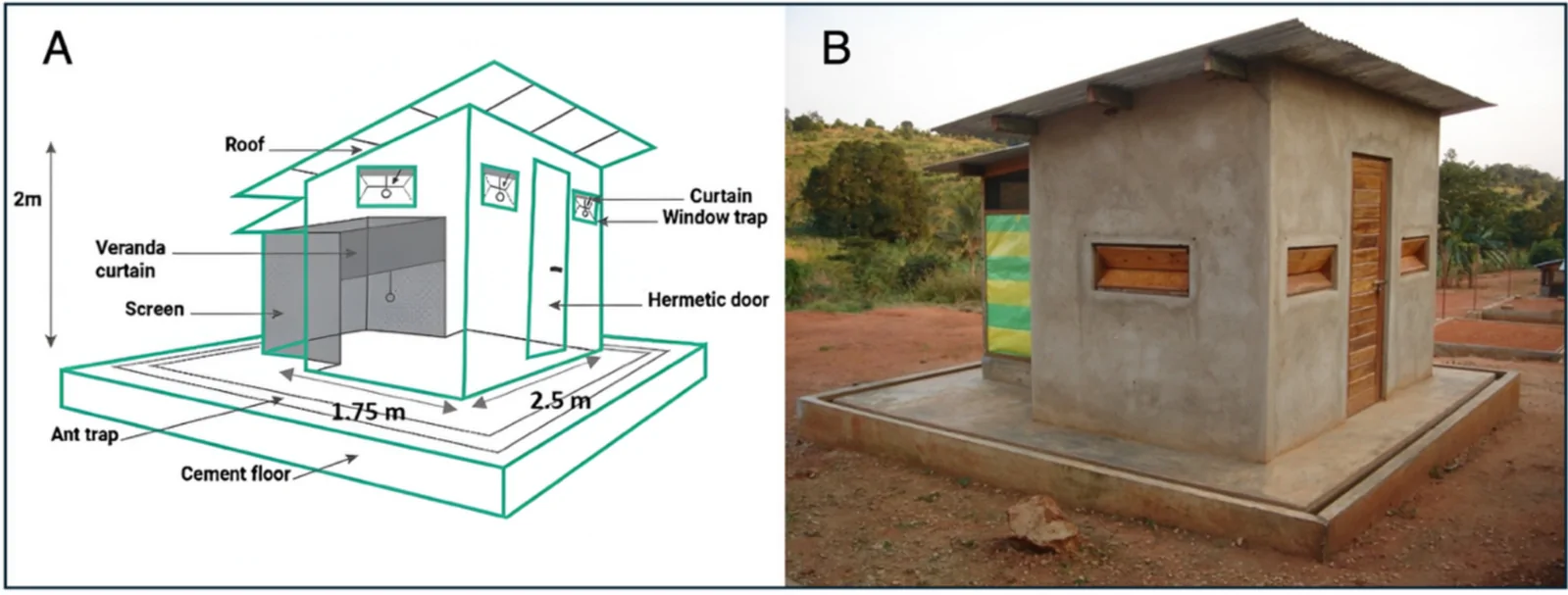
Schematic diagram (**A**) and photograph (**B**) of a West African-style hut (schematic from WHO prequalification of vector control products, 2023, p. 9, World Health Organization [40]) Photo: RM Oxborough, Zeneti experimental hut site, Tanzania

**Fig. 2:**
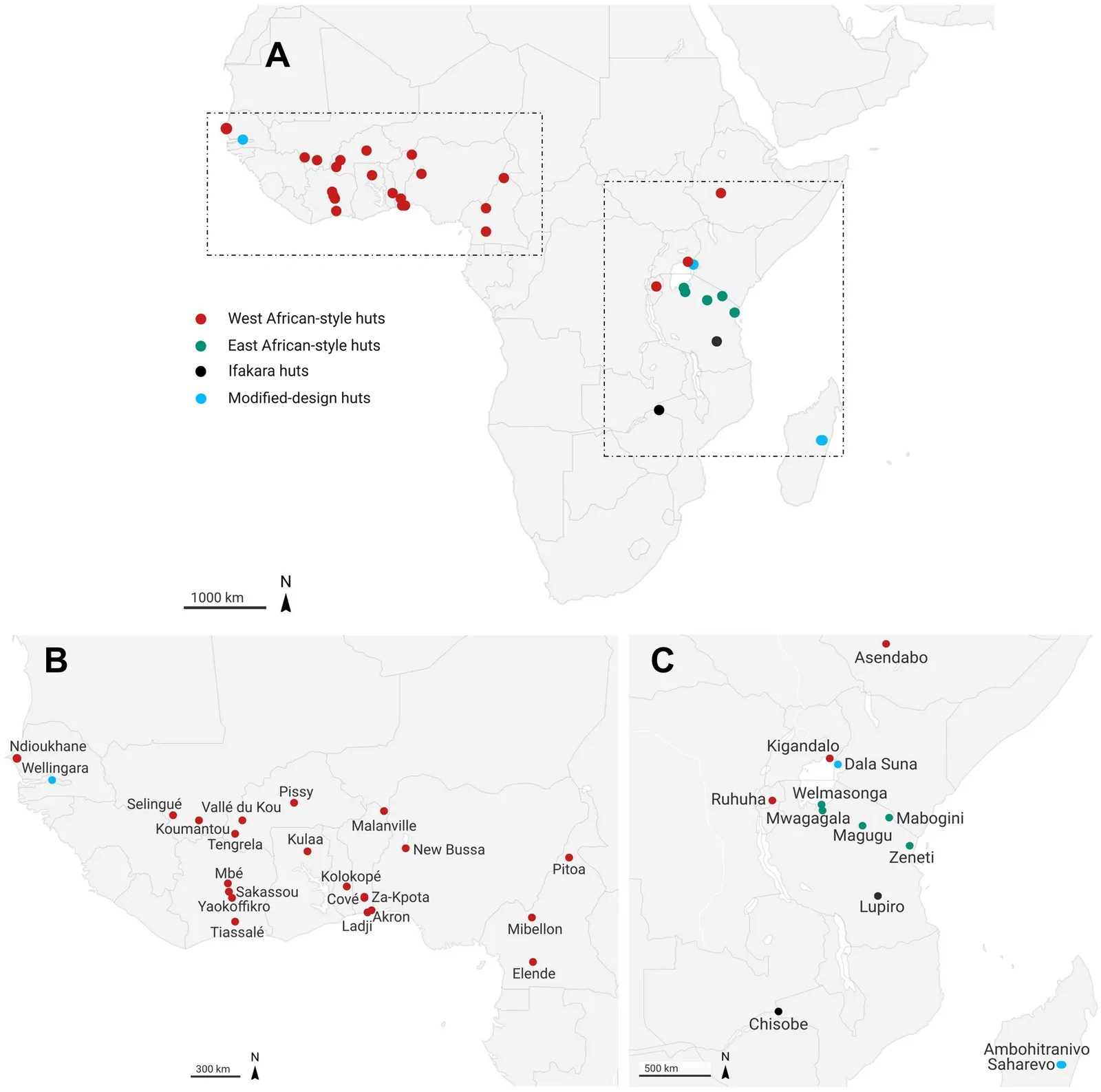
Map of Africa showing locations of West African-style, East African-style, Ifakara, and modified-design huts used from 2000 to 2025 for vector control product evaluation (**A**), with details of site locations for West Africa (**B**) and East Africa (**C**) (adapted from Nash et al. [39])

West African-style huts were originally based on the design of traditional houses in Burkina Faso but have since become the most widely adopted design in 24 of 34 (71%) experimental hut sites in West Africa and beyond (Cameroon, Ethiopia, Uganda, and Rwanda) (Table 1, Fig. 1). Our review documented 34 conventional experimental hut sites in 17 countries (representing 40% [17/42] of malaria-endemic countries in sub-Saharan Africa) (Fig. 2). Of these experimental hut sites, only a handful feature repeatedly in publications, with five countries (Benin, Burkina Faso, Cameroon, Côte d’Ivoire, and Tanzania) having a sustained track record with multiple experimental hut sites and studies [39].

**Table 1:** Summary of experimental hut sites featured in publications from 2000 to 2025 for vector control product evaluation (adapted from Nash et al. [39])

Country	Site	Type of huts	Mosquito species^a^	Surrounding area/insecticide use	Cotton	Rice	Institution	References
Benin	Za-Kpota	W	*An. coluzzii*, *An. gambiae* s.s.	Rice and vegetables, significant pesticide use	−	X	University of Abomey-Calavi	[50]
	Cové	W	*An. coluzzii*	Situated at the center of a large rice-growing field	−	X	London School of Hygiene and Tropical Medicine (LSHTM)/Centre de Recherche Entomologique de Cotonou (CREC) Collaborative Centre	[51]
	Akron	W	*An. coluzzii*	Horticultural area in outskirts of Porto Novo	ND	ND	CREC	[52]
	Ladji	W	*An. coluzzii*	Village on outskirts of Cotonou, floods during the rainy season	−	−	CREC	[53]
	Malanville	W	*An. coluzzii*	In an irrigated rice-growing valley	−	X	CREC	[54]
Burkina Faso	Vallé du Kou	W	*An. coluzzii*	Insecticides used in surrounding rice fields and extensively on cotton	X	X	Institut de Recherche en Sciences de la Sante/Centre Muraz	[55]
	Pissy	W	*An. gambiae* s.l.	Situated near an artificial water dam	ND	ND	Centre National de Recherche et de Formation sur le Paludisme (CNRFP)	[56]
	Tengrela	W	*An. coluzzii*	Not described	ND	ND	CNRFP	[57]
Cameroon	Pitoa	W	*An. arabiensis*	Extensive cotton cultivation (around 35,000 ha.)	X	−	Not described	[54]
	Elende	W	*An. funestus*	Peri-urban locality near Yaoundé. Equatorial forest, degraded for farming	X	X	Centre for Research in Infectious Diseases (CRID)	[58]
	Mibellon	W	*An. funestus*	Mountainous zone near permanent water bodies which are breeding sites for *An. funestus*	X	X	CRID	[59]
Côte d’Ivoire	M’Bé	W	*An. coluzzii*	Next to rice fields, which provide extensive breeding sites for mosquitoes	−	X	Institut Pierre Richet	[60]
	Yaokoffikro	W	*An. gambiae* s.s.	Situated along a large valley producing rice and vegetables	−	X	Institut Pierre Richet	[61]
	Tiassalé	W	*An. coluzzii*	Rice paddies provide extensive breeding sites for mosquitoes	−	X	Centre Suisse de Recherches Scientifiques (CRSS)	[62]
	Sakassou	W	*An. gambiae* s.l.	Located 500 m from a large rice-growing area	−	X	CRSS	[63]
Ethiopia	Asendabo	W	*An. arabiensis*	Located 0.3 km from Gilgel Gibe hydroelectric power dam in Nada shore	−	−	Jimma University	[64]
The Gambia	Wellingara	Mo	*An. coluzzii*	Located close to a large area of irrigated rice	−	X	MRC Unit The Gambia	[65]
Ghana	Kulaa	W	*An. gambiae* s.s.	Rural northern savanna zone	−	−	University of Ghana	[66]
Kenya	Dala Suna	Mo	*An. funestus*	Shores of Lake Kanyaboli close to swamp habitats for malaria vectors	−	−	Centre for Global Health Research, Kenya Medical Research Institute	[67]
Madagascar	Saharevo/Ambohitranivo	Mo	*An. coustani, An. squamosus*	*Anopheles* densities heavily dependent on rice planting cycles	−	X	Institut Pasteur de Madagascar	[68]
Mali	Koumantou	W	*An. gambiae* s.l.	Characterized by cotton and high insecticide use	X	−	Malaria Research and Training Center (MRTC)	[69]
	Selingué	W	*An. gambiae* s.l.	Rice field area with low insecticide use	−	X	MRTC	
Nigeria	New Bussa	W	*An. gambiae* s.s.	Huts built facing perennial mosquito breeding sites	−	−	Nigerian Institute of Medical Research	[70]
Rwanda	Ruhuha	W	NA (release experiments only)	Not described	ND	ND	The Rwanda Biomedical Center	[71]
Senegal	Ndioukhane	W	NA (bioassay only)	Not described	ND	ND	Laboratory of Vector and Parasite Ecology of Cheikh, Anta Diop University	[72]
Tanzania	Zeneti	E	*An. gambiae* s.s., *An. funestus*	Huts built on the edge of a village with small farms	−	−	National Institute for Medical Research (NIMR) Amani Centre	[73]
	Mabogini	E	*An. arabiensis*	*Anopheles arabiensis* densities were heavily dependent on rice cropping cycles	−	X	Kilimanjaro Christian Medical University College (KCMUCo)	[74]
	Lupiro	I	*An. arabiensis*	Located between perennial irrigated rice fields (main larval mosquito habitat) and human settlements	−	X	Ifakara Health Institute (IHI)	[30]
	Magugu	E	*An. arabiensis*	Economic activities include livestock keeping and maize and rice farms	−	X	Tropical Pesticides Research Institute (TPRI)	[75]
	Mwagagala	E	*An. gambiae* s.s., *An. arabiensis*, *An. funestus*	Large rain-fed rice irrigation scheme which provides potential breeding sites for malaria mosquito vectors	−	X	NIMR Mwanza Center	[58]
	Welamasonga	E	*An. gambiae* s.s., *An. arabiensis*, *An. funestus*	Open fields used for cultivating rice and vegetables, with irrigation from ponds	−	X	NIMR Mwanza Center	[28]
Togo	Kolokopé	W	*An. coluzzii*, *An. gambiae* s.s.	Cotton cultivation site 236 ha. Pyrethroid-based insecticides used to spray fields	X	−	Universite de Lomé	[76]
Uganda	Kigandalo	W	*An. funestus, An. gambiae* s.l.	Rice fields, sugar cane plantations, permanent streams, and a low water table	−	X	Uganda Virus Research Institute	[77]
Zambia	Chisobe	I	*An. arabiensis*	Not described	−	−	National Malaria Elimination Centre	[78]

W, E, and I huts were also constructed for comparison studies in Zeneti, Tanzania [47] and W, I in Porto Novo, Benin [79]

Key: W, West African-style hut; E, East African-style hut; I, Ifakara hut; Mo, modified-design hut; ND, not described; X, present; −, absent

^a^Mosquito species refers to the most commonly collected malaria vector species (*An*. = *Anopheles*)

Experimental hut trial site locations within countries are often chosen based on several pragmatic criteria, including vector productivity and proximity to local research institutions for oversight and supervision. A large proportion (19/34) are located near rice-growing fields for consistent vector yield, with 6/34 near cotton fields (Table 1). A recent meta-analysis determined that malaria vector densities were six times higher in rice-growing than non-rice-growing areas, with ecological conditions of the early stages of rice fields preferred by larvae of *Anopheles gambiae* sensu lato (s.l.) [50]. While there has been a substantial increase in rice-growing areas in sub-Saharan Africa, with an estimated 16.6 million ha. cultivated in 2020, rice paddies are still an atypical environment and account for less than 0.2% of the total land area in sub-Saharan Africa [80, 81].

## Results

### PEHT prototype development

**Fig. 3:**
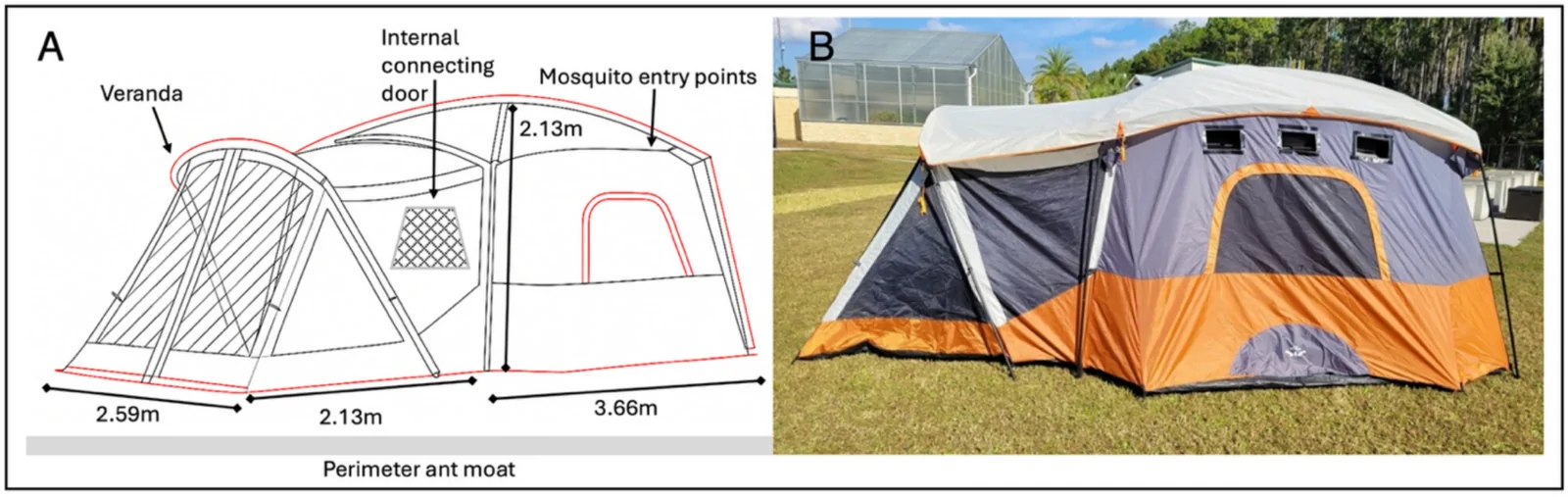
Schematic diagram (**A**) and photograph (**B**) of a portable experimental hut tent (PEHT). Photo: J Stamey, Anastasia Mosquito Control District, FL, USA

**Fig. 4:**
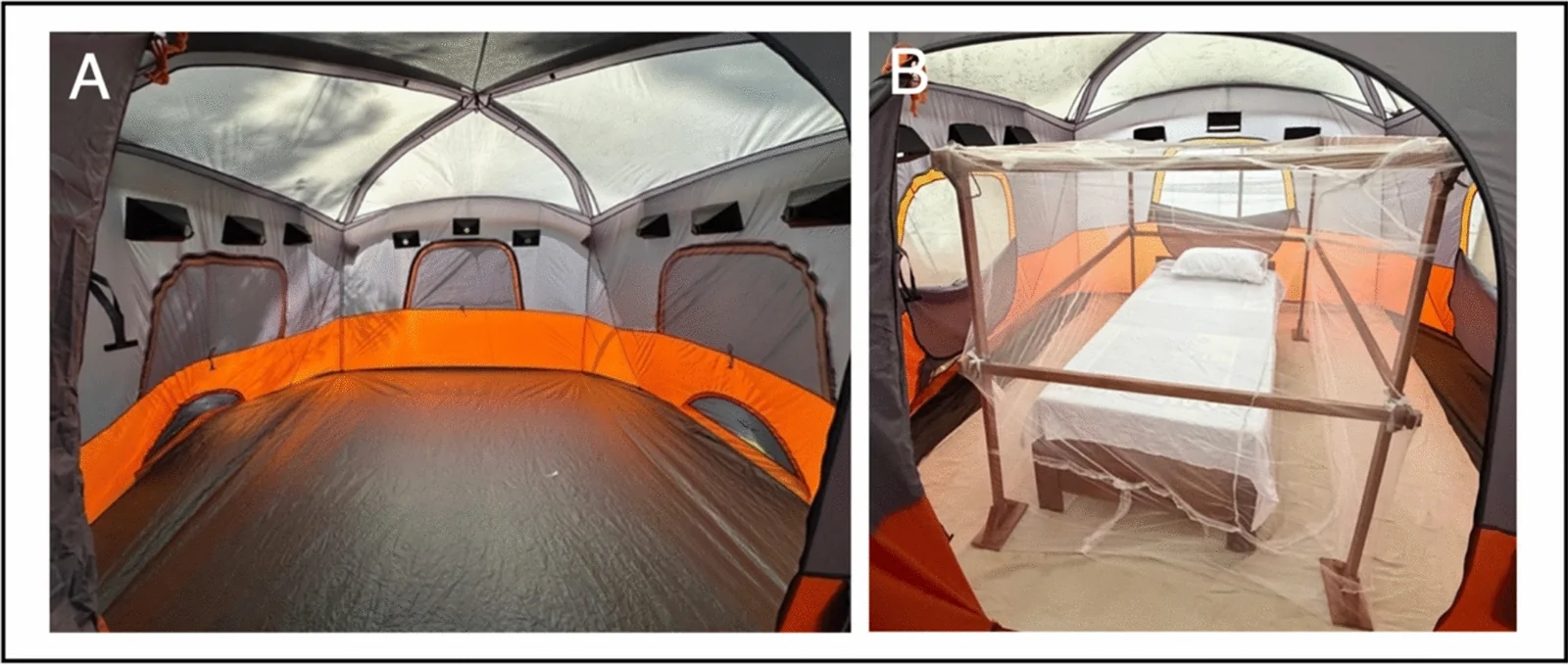
Interior view of PEHT fitted with nine 3D-printed entry points, bed, and mosquito net. Photos: T Wickramasinghe, University of Nevada, Las Vegas (UNLV), NV, USA (left) and University of Ghana (right)

The PEHT design (Fig. 3) is based on West African-style huts, by using large cabin-style tents and 3D printing technology to manufacture standardized, lightweight mosquito entry points. Traditional fixed West African-style huts measure 2.5 m (8.20 ft) length (L) × 1.75 m (5.74 ft) width (W) × 2 m (6.56 ft) height (H), with an internal volume of 8.75 m^3^, and are made from concrete bricks with a corrugated iron roof and a ceiling of polythene sheeting. Mosquitoes can enter the huts through four window slits (60 cm × 33 cm) with a 1 cm opening. A verandah trap made of polythene sheeting and screening mesh (2 m [L] × 1.5 m [W] × 1.5 m [H]) is fitted at the back of each hut [29]. PEHTs consist of a commercially available cabin tent (CORE^®^ Equipment, KS, USA) made from 68-denier polyethylene terephthalate (PET), measuring 3.66 m (12 ft) (L) × 3.05 m (10 ft) (W) × 2.13 m (7 ft) (H), with an internal volume of 23.8 m^3^. The PEHT is roughly 1.5 times longer and 1.7 times wider, is marginally taller, and has a 2.7-fold greater internal volume than the West African-style hut. As with the West African-style hut, there is a veranda trap, which measures 2.13 m (7 ft) (L) × 2.59 m (8.5 ft) (W). The veranda trap is connected to the PEHT via a zipped internal door, which is left partially open at night to allow mosquitoes to exit into the veranda and is sealed in the morning. A large tent model was intentionally chosen to accommodate a bed, a freestanding frame for a mosquito net, and sufficient space to inspect the area for dead and live mosquitoes. Durable white drop cloths can be placed on the floor of the PEHT and veranda to assist with visibility of dead mosquitoes. A single bed is positioned at the center of the PEHT, with a mosquito net hung over a supporting frame (Fig. 4). The net frame improves standardization compared to tucking nets under bed mattresses, which can be inadvertently contacted by sleepers during the night.

**Fig. 5:**
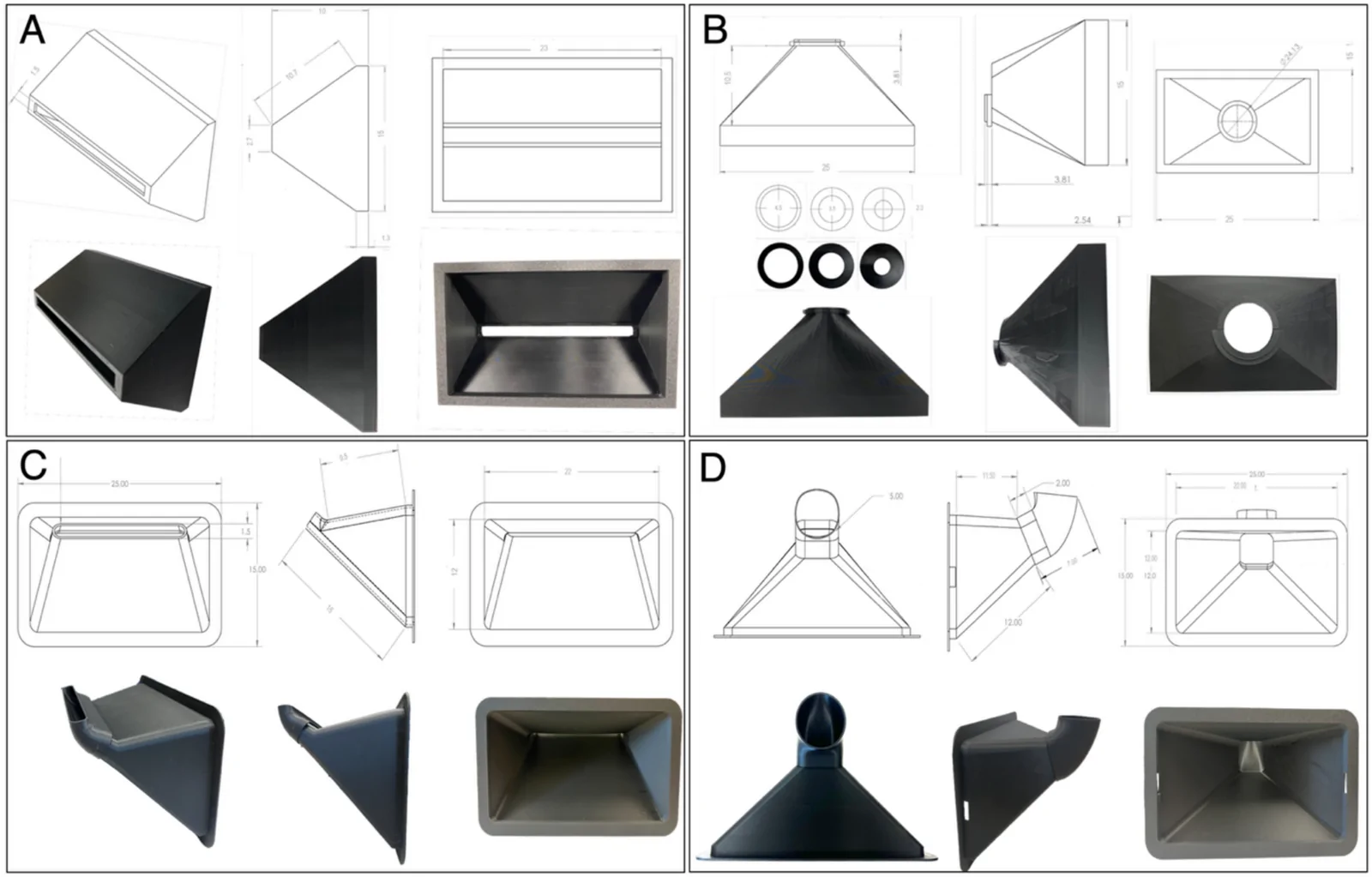
Schematic design (dimensions in cm) and photographs of four 3D-printed entry point designs: slit design (**A**), adjustable funnel (**B**), baffled slit (**C**), baffled funnel (**D**)

Mosquitoes can enter PEHTs through nine entry points measuring 25 cm (L) × 15 cm (W) × 10–12 cm (D). The length of 25 cm was chosen as the maximum for a standard low-cost 3D printer. The prototype parts were printed using a Bambu Lab 3D printer (model A1, Shenzhen, China). We produced four designs of mosquito entry points that were 3D-printed using lightweight 1.75 mm black polylactic acid (PLA) plastic filament. The four mosquito entry point designs are shown in Fig. 5 and will be optimized and validated during future release–recapture experiments, to achieve maximal *Anopheles* entry while restricting escape. Three mosquito entry points are positioned on each of three sides of the PEHT (Fig. 4), with openings created using a soldering iron to prevent frayed ends. Two initial designs of 3D-printed mosquito entry points were attached with Velcro strips, while two subsequent designs are attached using a 3D-printed retainer, reinforced with mini binder clips.

An important feature of PEHTs is portability, so that studies of vector control tools can be conducted in remote, high-burden settings. Each tent weighs approximately 16 kg (36 lbs.) and can be packed into a standard 27-gallon (approximately 100 L) storage container, allowing for secure transport using a pickup truck. PEHTs can be installed quickly, followed by erection of a shade tarpaulin (to protect tent material from damaging ultraviolet (UV) rays and to prevent extreme temperatures), and the preparation of a temporary ant exclusion moat. PEHTs will be optimized in the future using semi-field screenhouses for release–recapture experiments and characterized with reference to West African-style huts, before use for field assessments of vector control products. The approximate cost of a PEHT is $475, consisting of 11-person tent ($228 wholesale price), 3D-printed mosquito entry points ($72; $8 per entry point), perimeter ant treatment ($35), shade tarpaulin ($60), and temporary ant barrier ($80), not including standard accessories (bed frame, white floor sheets). Although cost is relevant, the primary impetus for PEHTs is portability across a range of operational settings.

## Discussion

### Potential utilization of PEHTs

Vector control decisions must be tailored based on contextual factors and are best addressed through experimental hut studies against local mosquitoes, which can have distinct behavioral traits, insecticide resistance phenotypes, and underlying resistance mechanisms. A major advantage of PEHTs is that they can be utilized within high-malaria-burden villages for the evaluation of field-aged ITNs from communities against local malaria vector populations, to support data-driven vector control decision-making by NMC/EPs. PEHTs can be deployed in areas of greatest burden, where limited fixed experimental hut site capacity exists, such as Nigeria and the Democratic Republic of the Congo, which accounted for > 37% (104/282 million) of malaria cases in Africa in 2024 [82], to answer subnational operational vector control questions. A critical decision that NMC/EPs must address is which type of ITN is most cost-effective in each region. This is particularly relevant, as PBO and CFP content tends to decrease faster than partner PYs during net use [10]. Understanding the operational performance of different ITN classes in the community as they begin to age in different societal and environmental contexts is paramount to optimizing the choice of ITN class and frequency of distribution [26]. Entomological data from PEHTs characterizing the bio-efficacy of the net for killing and repelling local mosquitoes, and how ITN impact changes over time, will be included within a mechanistic model of *Plasmodium falciparum* malaria transmission dynamics [83]. Simple-to-use online tools can allow public health professionals to combine PEHT data with local entomological, epidemiological, and product cost information to generate bespoke estimates of the number of malaria cases averted for each type of ITN. This will allow exploration of the cost-effectiveness of different vector control tool options under distinct scenarios and enable locally driven evidence-based decision-making considering regional preferences and budgetary restrictions [84].

There are inevitably limitations with the PEHT system. IRS cannot be easily evaluated (although IRS proxies such as treated wall hangings [85] or durable wall lining [86] could potentially be evaluated). Contamination of the inner tent material with insecticide-treated materials (e.g., when hanging ITNs) must be avoided by adherence to strict protocols and decontamination using mild detergents and airing out tents between intervention rotation. Mosquito resting behavior could potentially be altered by the polyester walls and ceiling being atypical compared to materials used in typical rural homes (e.g., mud, concrete walls, thatch ceilings). Studies are needed to compare mosquito behavior in PEHTs compared to conventional West African-style huts. Long-term PEHT durability can be enhanced by reinforcement of seams and proper care (e.g., avoiding UV light, careful packing in protective boxes when not in use). Nevertheless, PEHTs should be seen as temporary tools, just as mosquito surveillance traps are replaced on a periodic basis. Tents would need to be imported with associated shipping and clearance costs, as is commonly done for other items not available within Africa such as mosquito traps and molecular reagents. A potential risk is that the chosen tent design may become obsolete; however, given the wide range of cabin tents available, similar models can be readily substituted, and validation experiments will test alternative models to determine whether results are comparable.

Beyond the evaluation of ITNs, PEHTs can be utilized for the monitoring and evaluation of novel vector control technologies, such as spatial repellent (SR) emanators and attractive targeted sugar baits (ATSB). Transfluthrin SR emanators have generated much optimism following a cRCT in Kenya that showed 32.1% protective efficacy from malaria infection [87]. It is vitally important to monitor the effectiveness of SR emanators over time, particularly as they rely on PYs to which behavioral resistance has already been detected [88]. PEHTs can be utilized within villages where SR emanators have been distributed to monitor deterrence of mosquito entry, repellency, blood-feeding success, and mortality so that any loss of efficacy is detected. ATSB have been evaluated outside of houses in large-scale cRCTs in Mali, Zambia, and Kenya, but with limited epidemiological evidence of improved malaria control [89–91]. Nevertheless, several studies have indicated that ATSB may be better suited to either indoor use or combined indoor/outdoor deployment [92–94]. A series of studies is needed to optimize the deployment modality of ATSB and visualize frequency of feeding on sugar baits, which can be effectively studied in PEHTs [95].

The portable nature of PEHTs allows for utilization in unstable locations such as ongoing humanitarian emergencies, military operating sites, or where invasive species are detected. A recent WHO-funded meta-analysis concluded that there is limited evidence for most vector control tools during humanitarian emergencies [96]. The portable nature of PEHTs is a particular advantage, as they can be erected in refugee camps for short-term evaluation of singular or combination vector control strategies. Better vector control decision-making during humanitarian emergencies can have a substantial benefit for the most vulnerable communities in society [97]. Vector-borne diseases remain a major health threat to armed forces deployed in the tropics, with evidence-based vector control and personal protection strategies essential for operational readiness of armed forces [98]. PEHTs can be used to guide vector control decision-making at military bases and remote outposts. The portable nature of PEHTs also facilitates utilization in locations where invasive species are detected, such as *Anopheles stephensi* Liston, 1901, which has spread throughout the Horn of Africa [99]. Since *An. stephensi* was implicated in malaria resurgence in Djibouti [100] and parts of Ethiopia [101], vital questions remain regarding the most cost-effective methods of *An. stephensi* vector control [102]. PEHTs could be valuable in determining the relative efficacy of indoor interventions against *An. stephensi*, particularly in areas of malaria resurgence.

While our focus is on areas of greatest malaria burden in sub-Saharan Africa, PEHTs can be utilized on other continents to address local, specific questions, for example, assessing the performance of vector control tools (including regionally important tools such as insecticide-treated hammocks and topical repellents) in remote communities of Latin America [103], Southeast Asia [104], and Pacific island nations such as Papua New Guinea [105]. In such locations, outdoor biting is more common; nevertheless, PEHTs can provide invaluable data on mosquito species diversity and localized behavioral traits including house entry, indoor resting, exiting behavior, and blood-feeding success [106]. PEHTs may be especially useful in settings with emerging vector-borne disease threats. In the United States, for example, PEHTs could facilitate the monitoring of the bionomics and control of locally relevant vectors, including *Anopheles crucians* and *Anopheles quadrimaculatus*, which were implicated in recent autochthonous malaria transmission in Florida in 2003 and 2023 [107, 108]. The versatility of PEHTs also allows for evaluation of vector control tools against arbovirus vectors, such as day-biting *Aedes* or crepuscular *Culex*, with the possibility of baiting with artificial attractants [109].

## Conclusions

This study describes the innovative concept and design of PEHTs for expanded evaluation of malaria vector control tools in high-malaria-burden communities against their local, heterogeneous mosquito vector populations, to improve regional evidence-based vector control decision-making.

## Data Availability

Data supporting the main conclusions of this study are included in the manuscript.

## Abbreviations

ATSB: Attractive targeted sugar baits
CFP: Chlorfenapyr
cRCT: Cluster-randomized controlled trial
IRS: Indoor residual spraying
ITN: Insecticide-treated net
NMC/EP: National malaria control/elimination program
PBO: Piperonyl butoxide
PET: Polyethylene terephthalate
PLA: Polylactic acid
PEHT: Portable experimental hut tent
PY: Pyrethroid
SR: Spatial repellent
UV: Ultraviolet
WHO: World Health Organization
WHOPES: WHO Pesticide Evaluation Scheme

## Footnotes

1 “Dual-active ingredient” is an unhelpful terminology that does not distinguish between highly effective PY-CFP ITNs and PY-pyriproxyfen (PPF) ITNs, which perform no better epidemiologically than standard PY ITNs. Unfortunately, the Alliance for Malaria Prevention categorize both these ITNs as dual-AI, hence we are left to assume that most of these nets are PY-CFP ITNs, as there is no rationale for purchasing PY-PPF ITNs (see reference [4] for more information).
